# Assessing the occurrence of hypertension in patients receiving calcitonin gene-related peptide monoclonal antibodies for episodic and chronic migraine: a systematic review and meta-analysis

**DOI:** 10.22514/jofph.2024.036

**Published:** 2024-12-12

**Authors:** Meiqi Di, Lingling Hu, Shuhua Gui, Chaosheng Li, Likun Han

**Affiliations:** ^1^Department of Neurology, Affiliated Hospital of Jiangnan University, 214000 Wuxi, Jiangsu, China

**Keywords:** Headache, Migraine, CGRP monoclonal antibodies, Hypertension, Meta-analysis

## Abstract

Calcitonin gene-related peptide (CGRP) monoclonal antibodies in the treatment of episodic and chronic migraine was invetigated. A comprehensive literature search was conducted in Ovid Medline, Web of Science and Embase databases from their inception until April 2024 for randomized controlled trials comparing CGRP monoclonal antibodies with placebo or other active treatments in adults with episodic or chronic migraine. The primary outcome assessed was the incidence of hypertension, and secondary outcomes were tolerability, acceptability and adverse events. Data analysis was performed using a random-effects model, and the strength of evidence was evaluated using the Grading of Recommendation, Assessment, Development and Evaluation (GRADE) approach. A total of eleven studies involving 9729 participants were found eligible and included for data analysis. The results revealed that the pooled odds ratio for the incidence of hypertension in patients receiving CGRP monoclonal antibodies compared to placebo was (95% confidence interval (CI): 0.60, 2.21; *I*^2^ = 32%), suggesting no significant increase in hypertension risk. Moreover, no significant differences were observed in tolerability or acceptability between the CGRP monoclonal antibody and placebo groups. However, the overall risk of total adverse events was significantly higher in the CGRP monoclonal antibody group (odds ratio (OR): 1.13; 95% CI: 0.97, 1.33; *I*^2^ = 56%; *p* = 0.01). These findings indicate that CGRP monoclonal antibodies are well-tolerated and present a generally safe option for treating episodic and chronic migraine. Although there was no significant increase in the incidence of hypertension, a slight rise in overall adverse events was observed. Consequently, CGRP monoclonal antibodies may be considered a viable treatment option for patients who have not found other treatments effective or tolerable, or who have contraindications to alternative therapies. The study protocol was registered in the International Prospective Register of Systematic Reviews (PROSPERO) ( http://www.crd.york.ac.uk, registration number: CRD42024554897).

## 1. Introduction

Migraine is a prevalent neurological disorder affecting millions of individuals worldwide. It can be categorized into chronic migraine (CM) and episodic migraine (EM) based on the frequency of headache occurrences. According to the 2016 Global Burden of Disease Study, migraine is the second leading cause of global disability and the primary cause of disability among women aged 15–49 years [1]. Neurological disorders, including migraine, are increasingly recognized as major contributors to global death and disability [2, 3]. The pathophysiology of migraine is primarily associated with the activation of the trigeminovascular system and the release of calcitonin gene-related peptide (CGRP), a neuropeptide that plays a critical role in migraine pathogenesis [4]. Given the substantial burden of migraine and its impact on quality of life, the development of effective treatment options remains pivotal.

Current preventive treatments for migraine include monoclonal antibodies, such as erenumab, galcanezumab and fremanezumab, that can target the CGRP pathway. These pharmacologic agents have demonstrated efficacy in reducing the frequency and severity of migraine episodes [5, 6]. Specifically, randomized controlled trials (RCTs) have shown that erenumab significantly reduces the number of monthly migraine days in patients with EM when administered at doses of 70 or 140 mg over a 9–12 week period [4]. However, concerns have been raised about a potential association between these treatments and hypertension [7]. As cardiovascular safety is a critical consideration, it is important to assess the risks and benefits of these newly developed migraine treatments.

Given the need for both effective and safe migraine therapies, evaluating the cardiovascular safety profile of CGRP monoclonal antibodies is essential for patients with CM or EM, as defined by the International Classification of Headache Disorders, 3rd edition (ICHD-3Ed) criteria. This systematic review and meta-analysis aim to provide a comprehensive assessment of the cardiovascular safety associated with CGRP monoclonal antibodies. By synthesizing the available evidence, this study seeks to enhance the understanding of the risks and benefits of these medications, thereby informing clinical decision-making and improving patient care. The findings could be particularly relevant for healthcare professionals managing migraine patients and for researchers working to develop safer and more effective migraine treatments in the future.

## 2. Methods

This systematic review and meta-analysis adhered to the Preferred Reporting Items for Systematic Reviews and Meta-Analyses (PRISMA) guidelines [8], which were supplemented by an assessment tool for systematic reviews. The study protocol was registered in the International Prospective Register of Systematic Reviews (PROSPERO) (http://www.crd.york.ac.uk, registration number: CRD42024554897).

### 2.1 Literature search

A comprehensive literature search was conducted using the Ovid Medline, Web of Science and Embase databases from their inception until April 2024. The search strategy utilized the InterTASC Information Specialists’ Sub-Group (ISSG) Search Filters Resource [9], and detailed search strategies are provided in the **Supplementary material**. Additionally, reference lists from relevant systematic reviews and meta-analyses were manually reviewed to identify any additional pertinent studies.

### 2.2 Inclusion and exclusion criteria

Studies were considered included if they (1) were RCTs comparing CGRP monoclonal antibodies with placebo or other active treatments in adults (≥18 years) with episodic or CM, including gepants as a comparison group due to their shared mechanism of action in inhibiting the CGRP pathway; (2) reported at least one of the following outcomes: tolerability, acceptability, the incidence of hypertension, or the number of adverse events; and (3) were published in English. Titles and abstracts were independently screened by two reviewers, followed by full-text assessments for eligibility. Any discrepancies were resolved through mutual discussion or by consulting a third reviewer.

### 2.3 Data extraction

Data extraction for study characteristics, participant demographics, intervention details and outcome measures was performed independently by two reviewers using a standardized form. The risk of bias in the included RCTs was assessed using the Cochrane Risk of Bias 2 tool. Any discrepancies in data extraction and quality assessment were resolved through discussion or by involving a third reviewer.

### 2.4 Outcome measures

The primary outcome was the incidence of hypertension, and the secondary outcomes included tolerability (defined as the proportion of participants who did not discontinue the study due to adverse events), acceptability (defined as the proportion of participants who completed the study), and the number of adverse events in each group.

### 2.5 Data analysis

All analyses adhered to the intention-to-treat (ITT) principle. For each outcome, event proportions in each group were calculated, and odds ratios (ORs) with 95% confidence intervals (CIs) were estimated. Meta-analyses were conducted using a random-effects model with the Mantel-Haenszel method. Heterogeneity was assessed using the *I*^2^ statistic. Subgroup analyses were performed based on the type of CGRP monoclonal antibody and migraine diagnosis (episodic or chronic). Sensitivity analyses excluded studies with a high risk of bias. Publication bias was evaluated using funnel plots and Egger’s test. Statistical analyses were conducted using R software (version 4.0.3), with a two-sided *p*-value < 0.05 considered statistically significant. All analyses were also performed using RevMan Review Manager software version 5.4.1.

### 2.6 Grading the strength of evidence

The strength of evidence for each outcome was evaluated using the Grading of Recommendations, Assessment, Development and Evaluations (GRADE) approach. This assessment considered the risk of bias, inconsistency, indirectness, imprecision and publication bias. Evidence was categorized as high, moderate, low or very low.

## 3. Results

The initial literature search identified a total of 1360 records from Ovid Medline (n = 21 records), Embase (n = 1315 records), and Web of Science (n = 24 records). After removing 1063 duplicate records, 497 unique records remained for further assessment. An additional 20 duplicate articles were excluded during the full-text assessment, leaving 477 full-text articles for evaluation based on predefined inclusion and exclusion criteria. Of these, 462 studies were excluded due to lack of data on relevant outcomes, involvement of study types other than RCTs, or evaluation of unrelated interventions. Ultimately, 15 studies were included in the qualitative synthesis, and 11 studies were included in the quantitative synthesis for meta-analysis, following the exclusion of studies lacking necessary data (Fig. 1) [10, 11, 12, 13, 14, 15, 16, 17, 18, 19]. The risk of bias assessment (Fig. 2) showed that most studies had a low risk of bias, though a few domains raised some concerns or indicated a high risk of bias.

**Fig. 1.** Study flow diagram.

**Fig. 2.** Risk of bias.

### 3.1 Characteristics of the studies

The meta-analysis included a total of 9729 participants, with the largest study being Ashina 2022, which evaluated erenumab in 2682 participants with episodic or CM over 24 weeks. All studies assessed the tolerability and safety of CGRP monoclonal antibodies for treating episodic or CM (Table 1). The participants’ mean ages ranged from 39.9 to 46.8 years, and most studies included both episodic and CM patients. The interventions studied were AMG 301 (erenumab), erenumab, TEV-48125 (fremanezumab), telcagepant, zavegepant, LY2951742 (galcanezumab) and fremanezumab. All studies were RCTs comparing active treatments to placebo.

**Table 1.** Basic characteristics of included studies.

Source	Sample size	Age, mean, SD	Subtype	Study type	Intervention/Control	No. of Patients Intervention/Placebo	Quantity, dose	Route of administration	Treatment duration	Outcomes of interest assessed
Ashina 2021	343	42.5 (9.5) 41.8 (9.9)	Episodic or Chronic	RCT	AMG 301/Placebo	206/137	AMG 301 70 mg; Placebo	Subcutaneous injection	12 wk	Tolerability and safety
Ashina 2022	2682	41.6 (11.2) 41.8 (11.15)	Episodic or Chronic	RCT	Erenumab/Placebo	1400/1043	Erenumab 7 mg; Erenumab 21 mg; Erenumab 70 mg; Placebo	Subcutaneous injection	24 wk	Tolerability and safety
Bigal 2015	297	40.8 (12.5) 42.0 (11.6)	Episodic or Chronic	RCT	TEV-48125/Placebo	191/95	TEV-48125 225 mg; TEV-48125 675 mg; Placebo	Subcutaneous injection	28 d	Tolerability and safety
Connor 2009	1294	41.7 (11.3) 41.9 (11.9)	Episodic	RCT	Telcagepant/Placebo	929/365	Telcagepant 50 mg; Telcagepant 150 mg; Telcagepant 300 mg; Placebo	Oral	2 h	Tolerability and safety
Croop 2022	1673	41.1 (12.9) 39.9 (12.0)	Episodic	RCT	Zavegepant/Placebo	1253/420	Zavegepant 5 mg; Zavegepant 10 mg; Zavegepant 20 mg; Placebo	Nasal spray	2 h	Tolerability and safety
Dodick 2014	218	40.9 (11.4) 41.9 (11.7)	Episodic	RCT	LY2951742/Placebo	108/110	LY2951742 150 mg; Placebo	Subcutaneous injection	12 wk	Tolerability and safety
Ferrari 2019	838	45.9 (11.0) 46.8 (11.1)	Episodic or Chronic	RCT	Fremanezumab/Placebo	559/279	Fremanezumab 675 mg; Fremanezumab 225 mg; Placebo	Subcutaneous injection	12 wk	Tolerability and safety
Goadsby 2017	955	40.8 (11.2) 41.3 (11.2)	Episodic	RCT	Erenumab/Placebo	635/319	Erenumab 140 mg; Placebo	Subcutaneous injection	24 wk	Tolerability and safety
Reuter 2018	246	44.6 (10.5) 44.2 (10.6)	Episodic	RCT	Erenumab/Placebo	121/125	Erenumab 140 mg; Placebo	Subcutaneous injection	12 wk	Tolerability and safety
Skljarevski 2018	922	41.4 (11.0) 42.3 (11.3)	Episodic	RCT	Galcanezumab/Placebo	454/461	Galcanezumab 120 mg; Galcanezumab 240 mg; Placebo	Subcutaneous injection	6 mon	Tolerability and safety
Takeshima 2021	261	44.2 (8.5) 44.6 (9.3)	Episodic or Chronic	RCT	Erenumab/Placebo	130/131	Erenumab 70 mg; Placebo	Subcutaneous injection	24 wk	Tolerability and safety

*SD: standard deviation; RCT: randomized controlled trial.*

The routes of administration varied among the interventions, with subcutaneous injection being the most common (9 studies), followed by oral administration (1 study) and nasal spray (1 study). Treatment durations ranged from 2 hours for acute migraine treatments (telcagepant and zavegepant) to 24 weeks for preventive treatments (erenumab, fremanezumab and galcanezumab). The primary outcomes assessed across all studies were tolerability and safety.

Overall, the studies encompassed a diverse array of CGRP monoclonal antibodies, routes of administration, treatment durations and patient populations, offering a comprehensive dataset for evaluating the tolerability and safety of these treatments for episodic and CM.

### 3.2 Incidence of hypertension

The incidence of hypertension was reported in 8 studies, encompassing a total of 5251 patients. Data analysis showed that the pooled OR for hypertension in patients receiving CGRP monoclonal antibodies compared to placebo was 1.15 (95% CI: 0.60, 2.21; *I*^2^ = 32%; Fig. 3). Subgroup analyses, which were based on specific drugs and treatment durations, did not reveal significant differences in hypertension incidence between the intervention and placebo groups.

**Fig. 3.** The forest plot and funnel plot of incidence of hypertension, subgroup analysis by erenumab and treatment duration. CI: confidence interval; M-H: Mantel-Haenszel test; SE: standard Error; OR: odds ratio.

### 3.3 Tolerability and acceptability

Tolerability data were available from all studies, involving a total of 5465 patients. The pooled OR for tolerability in patients receiving CGRP monoclonal antibodies compared to placebo was 1.32 (95% CI: 0.82, 2.14; *I*^2^ = 0%; Fig. 4). Acceptability was reported in all studies as well, with the pooled OR for acceptability in patients receiving CGRP monoclonal antibodies compared to placebo being 0.92 (95% CI: 0.77, 1.11; *I*^2^ = 13%; Fig. 5).

**Fig. 4.** The forest plot and funnel plot of tolerability. CI: confidence interval; M-H: Mantel-Haenszel test; SE: standard Error; OR: odds ratio.

**Fig. 5.** The forest plot and funnel plot of acceptability. CI: confidence interval; M-H: Mantel-Haenszel test; SE: standard Error; OR: odds ratio.

### 3.4 Adverse events

All studies reported data on adverse events. The summary results indicated that the risk of total adverse events was significantly higher in the intervention group compared to placebo, with a pooled OR of 1.13 (95% CI: 0.97, 1.33; *I*^2^ = 56%; *p* = 0.01; Fig. 6).

**Fig. 6.** The forest plot and funnel plot of adverse events. CI: confidence interval; M-H: Mantel-Haenszel test; SE: standard Error; OR: odds ratio.

## 4. Discussion

This systematic review and meta-analysis demonstrate that CGRP monoclonal antibodies are generally well-tolerated and safe for the treatment of episodic and CM. The pooled data showed no significant difference in the incidence of hypertension, tolerability or acceptability between CGRP monoclonal antibodies and placebo. However, there was a slight increase in the risk of total adverse events associated with CGRP monoclonal antibodies compared to placebo.

Real-world studies provide additional context for these findings. For example, a study by Muñoz-Vendrell [20] identified an increased risk of hypertension among patients with pre-existing cardiovascular conditions, highlighting the necessity for blood pressure monitoring in this population. Another study reported a low overall risk of hypertension but noted an increased incidence of cardiovascular events in patients with a history of cardiovascular disease [21, 22]. These observations suggest that while RCTs provide controlled conditions for assessing drug safety, real-world settings may reveal additional risks that warrant careful consideration.

The decision to use CGRP monoclonal antibodies versus other migraine treatments should consider factors such as patient characteristics, migraine attack profiles, and the safety and efficacy of available options [23]. Our review supports the use of CGRP monoclonal antibodies as a safe and well-tolerated treatment for patients who have not found other treatments effective or tolerable, or who have contraindications to alternative therapies.

Concerns regarding the potential risk of hypertension with CGRP monoclonal antibodies have been reported in recent studies. A retrospective cohort study found that 23.3% of patients treated with erenumab experienced worsening blood pressure, suggesting the need for ongoing blood pressure monitoring [24]. Similarly, a prospective follow-up study observed increased systolic and diastolic blood pressure in patients receiving erenumab and fremanezumab, with some requiring antihypertensive medication [25]. However, a separate retrospective study indicated that although 5.7% of patients had a significant increase in blood pressure, the overall risk of hypertension with anti-CGRP monoclonal antibodies remained low [26]. Patients with pre-existing hypertension were more likely to experience significant blood pressure increases, emphasizing the importance of monitoring this patient subgroup [23, 27]. These findings underscore the need for careful blood pressure management in patients receiving CGRP monoclonal antibodies, particularly those with existing hypertension or cardiovascular risk factors.

One notable advantage of CGRP monoclonal antibodies is their non-vasoconstrictive mechanism of action [28], which is particularly important for individuals with a history of cardiovascular events or multiple vascular risk factors, as triptans and ergot alkaloids—commonly used for acute migraine treatment—are vasoactive and should be avoided in these populations [28]. Additionally, CGRP monoclonal antibodies may be a suitable option for patients with gastrointestinal, kidney or cardiac comorbidities that limit the use of Non-steroidal anti-inflammatory drugs (NSAIDs) [29, 30].

The adverse events assessed in this review were found to be associated with immediate exposure to CGRP monoclonal antibodies. However, long-term safety data on these agents remain limited, and further research is needed to evaluate the potential risks associated with prolonged use [30]. It is important to monitor patients for adverse events and adjust treatment plans as necessary. The selection between pharmacologic and non-pharmacologic treatments for migraine should consider patient preference, tolerability and contraindications. While non-pharmacologic treatments, such as neuromodulation devices, have demonstrated promise in improving various pain measures, their evidence base remains limited compared to pharmacologic interventions [31]. Additionally, cost and lack of insurance coverage may pose barriers for many patients who need to access these devices. Current guidelines advise against the use of opioids and butalbital-containing medications for acute migraine treatment due to their low efficacy and high risk of adverse events [32]. Herein, our review found limited or insufficient evidence supporting the use of opioids for migraine management, with opioids being associated with higher rates of adverse effects compared to other treatment options or placebo. Consequently, the recommendations against opioids in current clinical guidelines remain valid.

This systematic review and meta-analysis has several limitations that should be considered when interpreting the results. First, the studies included in the analysis varied in design, patient populations, and treatment durations, contributing to heterogeneity in some analyses. Notably, eptinezumab was not included due to a lack of relevant studies, highlighting a gap that future research should address. Although gepants were included to provide a broader context for CGRP pathway inhibition, the focus of the review was on monoclonal antibodies, and the analysis did not encompass all gepants or CGRP monoclonal antibodies. Further research is needed to fully explore these treatments. Second, the long-term safety and efficacy of CGRP monoclonal antibodies beyond the studied treatment durations remain uncertain. Additional research is required to evaluate the potential risks and benefits of prolonged use. Third, a majority of the included studies were funded by pharmaceutical companies, which may introduce a degree of bias. Fourth, the review did not assess the comparative effectiveness of CGRP monoclonal antibodies against other established migraine treatments. Future head-to-head trials are needed to determine their relative efficacy and safety. Finally, the cost-effectiveness of CGRP monoclonal antibodies was not evaluated, an important consideration for patients, healthcare providers and policymakers. Despite these limitations, the review offers valuable insights into the tolerability and safety of CGRP monoclonal antibodies for treating episodic and CM.

## 5. Conclusions

This systematic review and meta-analysis indicate that the use of CGRP monoclonal antibodies for treating episodic and CM does not result in a significant increase in the incidence of hypertension. Nevertheless, further research is necessary to evaluate the long-term cardiovascular safety of these medications and to identify any potential risk factors for developing hypertension during treatment.

## Supplementary Material



## References

[1] Disease GBD, Injury I, Prevalence C. Global, regional, and national incidence, prevalence, and years lived with disability for 328 diseases and injuries for 195 countries, 1990–2016: a systematic analysis for the Global Burden of Disease Study 2016. The Lancet. 2017; 390: 1211–1259. 10.1016/S0140-6736(17)32154-2 PMC5605509 28919117

[2] Collaborators GBDN. Global, regional, and national burden of neurological disorders, 1990–2016: a systematic analysis for the Global Burden of Disease Study 2016. The Lancet Neurology. 2019; 18: 459–480. 10.1016/S1474-4422(18)30499-X PMC6459001 30879893

[3] Petrun AM, Selinsek J, Mekis D. Neurological complication during pregnancy, delivery and puerperium requiring intensive therapy management. Signa Vitae. 2024; 20: 17–25.

[4] Datta A, Gupta S, Maryala S, Aggarwal V, Chopra P, Jain S. Erenumab for episodic migraine. Pain Management. 2022; 12: 587–594. 10.2217/pmt-2021-0077 35313740

[5] Mulleners WM, Kim BK, Láinez MJA, Lanteri-Minet M, Pozo-Rosich P, Wang S, *et al*. Safety and efficacy of galcanezumab in patients for whom previous migraine preventive medication from two to four categories had failed (CONQUER): a multicentre, randomised, double-blind, placebo-controlled, phase 3b trial. The Lancet Neurology. 2020; 19: 814–825. 10.1016/S1474-4422(20)30279-9 32949542

[6] Ferrari MD, Diener HC, Ning X, Galic M, Cohen JM, Yang R, *et al*. Fremanezumab versus placebo for migraine prevention in patients with documented failure to up to four migraine preventive medication classes (FOCUS): a randomised, double-blind, placebo-controlled, phase 3b trial. The Lancet. 2019; 394: 1030–1040. 10.1016/S0140-6736(19)31946-4 31427046

[7] Van Der Arend BWH, Van Veelen N, De Ruijter JET, Olsen MH, MaassenVanDenBrink A, Terwindt GM. Safety considerations in the treatment with anti-CGRP(R) monoclonal antibodies in patients with migraine. Frontiers in Neurology. 2024; 15: 1387044. 10.3389/fneur.2024.1387044 PMC11089895 38742048

[8] Stewart LA, Clarke M, Rovers M, Riley RD, Simmonds M, Stewart G, *et al*. Preferred Reporting Items for Systematic Review and Meta-Analyses of individual participant data: the PRISMA-IPD Statement. JAMA. 2015; 313: 1657–1665. 10.1001/jama.2015.3656 25919529

[9] Chen CH. Revision and validation of a scale to assess pregnancy stress. Journal of Nursing Research. 2015; 23: 25–32. 10.1097/jnr.0000000000000047 25226045

[10] Ashina M, Doležil D, Bonner JH, Zhou L, Klatt J, Picard H, *et al*. A phase 2, randomized, double-blind, placebo-controlled trial of AMG 301, a pituitary adenylate cyclase-activating polypeptide PAC1 receptor monoclonal antibody for migraine prevention. Cephalalgia. 2021; 41: 33–44. 10.1177/0333102420970889 PMC7786389 33231489

[11] Ashina M, Lanteri-Minet M, Pozo-Rosich P, Ettrup A, Christoffersen CL, Josiassen MK, *et al*. Safety and efficacy of eptinezumab for migraine prevention in patients with two-to-four previous preventive treatment failures (DELIVER): a multi-arm, randomised, double-blind, placebo-controlled, phase 3b trial. The Lancet Neurology. 2022; 21: 597–607. 10.1016/S1474-4422(22)00185-5 35716692

[12] Bigal ME, Dodick DW, Rapoport AM, Silberstein SD, Ma Y, Yang R, *et al*. Safety, tolerability, and efficacy of TEV-48125 for preventive treatment of high-frequency episodic migraine: a multicentre, randomised, double-blind, placebo-controlled, phase 2b study. The Lancet Neurology. 2015; 14: 1081–1090. 10.1016/S1474-4422(15)00249-5 26432182

[13] Connor KM, Shapiro RE, Diener HC, Lucas S, Kost J, Fan X, *et al*. Randomized, controlled trial of telcagepant for the acute treatment of migraine. Neurology. 2009; 73: 970–977. 10.1212/WNL.0b013e3181b87942 PMC2754336 19770473

[14] Croop R, Madonia J, Stock DA, Thiry A, Forshaw M, Murphy A, *et al*. Zavegepant nasal spray for the acute treatment of migraine: a Phase 2/3 double-blind, randomized, placebo-controlled, dose-ranging trial. Headache. 2022; 62: 1153–1163. 10.1111/head.14389 PMC9827820 36239038

[15] Dodick DW, Goadsby PJ, Spierings ELH, Scherer JC, Sweeney SP, Grayzel DS. Safety and efficacy of LY2951742, a monoclonal antibody to calcitonin gene-related peptide, for the prevention of migraine: a phase 2, randomised, double-blind, placebo-controlled study. The Lancet Neurology. 2014; 13: 885–892. 10.1016/S1474-4422(14)70128-0 25127173

[16] Goadsby PJ, Reuter U, Hallstrom Y, Broessner G, Bonner JH, Zhang F, *et al*. A controlled trial of erenumab for episodic migraine. The New England Journal of Medicine. 2017; 377: 2123–2132. 10.1056/NEJMoa1705848 29171821

[17] Reuter U, Lucas C, Dolezil D, Hand AL, Port MD, Nichols RM, *et al*. Galcanezumab in patients with multiple previous migraine preventive medication category failures: results from the open-label period of the CONQUER trial. Advances in Therapy. 2021; 38: 5465–5483. 10.1007/s12325-021-01911-7 PMC8523004 34542830

[18] Skljarevski V, Matharu M, Millen BA, Ossipov MH, Kim BK, Yang JY. Efficacy and safety of galcanezumab for the prevention of episodic migraine: results of the EVOLVE-2 Phase 3 randomized controlled clinical trial. Cephalalgia. 2018; 38: 1442–1454. 10.1177/0333102418779543 29848108

[19] Takeshima T, Sakai F, Hirata K, Imai N, Matsumori Y, Yoshida R, *et al*. Erenumab treatment for migraine prevention in Japanese patients: Efficacy and safety results from a Phase 3, randomized, double-blind, placebo-controlled study. Headache. 2021; 61: 927–935. 10.1111/head.14138 PMC8361926 34153117

[20] Muñoz-Vendrell A, Campoy S, Caronna E, Alpuente A, Torres-Ferrus M, Nieves Castellanos C, *et al*. Effectiveness and safety of anti-CGRP monoclonal antibodies in patients over 65 years: a real-life multicentre analysis of 162 patients. The Journal of Headache and Pain. 2023; 24: 63. 10.1186/s10194-023-01585-2 PMC10236648 37268904

[21] Barbanti P, Aurilia C, Egeo G, Proietti S, D’Onofrio F, Torelli P, *et al*. Ultra-late response (>24 weeks) to anti-CGRP monoclonal antibodies in migraine: a multicenter, prospective, observational study. Journal of Neurology. 2024; 271: 2434–2443. 10.1007/s00415-023-12103-4 PMC11055785 38231271

[22] Barbanti P, Aurilia C, Egeo G, Proietti S, Torelli P, d’Onofrio F, *et al*. Impact of multiple treatment cycles with anti-CGRP monoclonal antibodies on migraine course: focus on discontinuation periods. Insights from the multicenter, prospective, I-GRAINE study. Journal of Neurology. 2024; 271: 2605–2614. 10.1007/s00415-024-12192-9 PMC11055727 38342785

[23] Aggarwal P, Goyel V, Mathur S, Sachdev V. Effect of stainless-steel crown and preformed zirconia crown on the periodontal health of endodontically treated primary molars correlating with IL-1β: an *in vivo* study. Journal of Clinical Pediatric Dentistry. 2022; 46: 199–203. 10.17796/1053-4625-46.3.5 35830632

[24] Chhabra N, Mead-Harvey C, Dodoo CA, Iser C, Taylor H, Chaudhary H, *et al*. Blood pressure elevation in erenumab-treated patients with migraine: a retrospective real-world experience. Headache. 2024; 64: 233–242. 10.1111/head.14679 38411625

[25] de Vries Lentsch S, van der Arend BWH, Maassen VanDenBrink A, Terwindt GM. Blood pressure in patients with migraine treated with monoclonal Anti-CGRP (Receptor) antibodies: a prospective follow-up study. Neurology. 2022; 99: e1897–e1904. 10.1212/WNL.0000000000201008 PMC9620812 36195452

[26] Guerzoni S, Castro FL, Brovia D, Baraldi C, Pani L. Evaluation of the risk of hypertension in patients treated with anti-CGRP monoclonal antibodies in a real-life study. Neurological Sciences. 2024; 45: 1661–1668. 10.1007/s10072-023-07167-z 37926748

[27] Anderson EM, Luu M, Kamrava M. Pathologic primary tumor factors associated with risk of pelvic and paraaortic lymph node involvement in patients with endometrial adenocarcinoma. European Journal of Gynaecological Oncology. 2023; 44: 37–42.

[28] Falkenberg K, Bjerg HR, Olesen J. Two-hour CGRP infusion causes gastrointestinal hyperactivity: possible relevance for CGRP antibody treatment. Headache. 2020; 60: 929–937. 10.1111/head.13795 32227602

[29] Ailani J, Kaiser EA, Mathew PG, McAllister P, Russo AF, Vélez C, *et al*. Role of calcitonin gene-related peptide on the gastrointestinal symptoms of migraine-clinical considerations: a narrative review. Neurology. 2022; 99: 841–853. 10.1212/WNL.0000000000201332 PMC9651456 36127137

[30] Favoni V, Giani L, Al-Hassany L, Asioli GM, Butera C, de Boer I, *et al*. CGRP and migraine from a cardiovascular point of view: what do we expect from blocking CGRP? The Journal of Headache and Pain. 2019; 20: 27. 10.1186/s10194-019-0979-y PMC6734543 30866804

[31] Tassorelli C, Diener HC, Silberstein SD, Dodick DW, Goadsby PJ, Jensen RH, *et al*. Guidelines of the international headache society for clinical trials with neuromodulation devices for the treatment of migraine. Cephalalgia. 2021; 41: 1135–1151. 10.1177/03331024211010413 33990161

[32] Hamilton KT, Robbins MS. Migraine treatment in pregnant women presenting to acute care: a retrospective observational study. Headache. 2019; 59: 173–179. 10.1111/head.13434 30403400

